# Prevalence of tuberculosis, COVID-19, chronic conditions and vulnerabilities among migrants and refugees: an electronic survey

**DOI:** 10.1590/1518-8345.5928.3690

**Published:** 2023-02-10

**Authors:** Sonia Vivian de Jezus, Carolina Maia Martins Sales, Silvia das Dores Rissino, Helaine Jacinta Salvador Mocelin, Mariana Pereira da Silva Araújo, Ricardo Alexandre Arcêncio, Vania Maria Silva Araújo, Nahari de Faria Marcos Terena, Paula de Souza Silva Freitas, Ethel Leonor Noia Maciel

**Affiliations:** 1 Universidade Federal do Espírito Santo, Centro de Ciências da Saúde, Vitória, ES, Brazil.; 2 Universidade Federal do Espírito Santo, Centro Universitário Norte do Espírito Santo (CEUNES), São Mateus, ES, Brazil.; 3 Universidade de São Paulo, Escola de Enfermagem de Ribeirão Preto, PAHO/WHO Collaborating Centre for Nursing Research Development, Ribeirão Preto, SP, Brazil.; 4 Rede Brasileira de Pesquisas em Tuberculose REDE-TB, Parque Tecnológico da Universidade Federal do Rio de Janeiro, Rio de Janeiro, RJ, Brazil.; 5 Universidade de Roma La Sapienza, Departamento de Estatística, Roma, Vaticano, Italia.

**Keywords:** Emigration and Immigration, Tuberculosis, Health Vulnerability, Brazil, Coronavirus, Epidemiology, Migração Internacional, Tuberculose, Vulnerabilidade em Saúde, Brasil, Coronavirus, Epidemiologia, Emigración e Inmigración, Tuberculosis, Vulnerabilidad en Salud, Brasil, Coronavirus, Epidemiología

## Abstract

**Objective:**

to analyze the prevalence of tuberculosis, coronavirus, chronic conditions and vulnerabilities among migrants and refugees in Brazil.

**Method:**

this is a cross-sectional study of the electronic survey type conducted with international migrants during the COVID-19 pandemic. Descriptive statistics was applied for the analysis, with calculation of position and dispersion measures. Regarding the categorical variables, relative and absolute frequencies were estimated.

**Results:**

the study participants were 553 migrants and refugees, verifying 3.07%, 7.2% and 27.3% prevalence of tuberculosis, COVID-19 and chronic conditions, respectively. Among the vulnerabilities, 32% reported unemployment, 37.6% moved to Brazil as a result of the social situation in their countries and 33.6% were living as refugees or sheltered people.

**Conclusion:**

tuberculosis, chronic diseases and COVID-19 presented higher prevalence values in migrants and refugees than in the general population. As this is a population group that still has significant difficulty accessing health services and social protection systems, based on diverse evidence, the study will subsidize public policies, Nursing care and the incorporation of new routines in the service.

## Resumo

**Objetivo:**

analisar a prevalência de tuberculose, coronavírus, condições crônicas e vulnerabilidades entre migrantes e refugiados no Brasil.

**Método:**

trata-se de estudo transversal, do tipo inquérito eletrônico, realizado com migrantes internacionais durante a pandemia de COVID-19. Para a análise, aplicou-se estatística descritiva, com cálculo de medidas de posição e de dispersão. Quanto às variáveis categóricas, estimaram-se as frequências relativas e absolutas.

**Resultados:**

participaram do estudo 553 migrantes e refugiados, verificando-se prevalência de 3,07% de tuberculose, 7,2% de COVID-19 e 27,3% de condições crônicas. Entre as vulnerabilidades, 32% referiram desemprego, 37,6% mudaram para o Brasil em decorrência da situação social do seu país e 33,6% residiam em asilo e ou abrigo.

**Conclusão:**

a tuberculose, as doenças crônicas e a COVID-19 apresentaram maior prevalência em migrantes e refugiados que na população em geral. Por tratar-se de uma população ainda com grande dificuldade de acesso aos serviços de saúde e aos sistemas de proteção social, o estudo subsidiará, com base em evidências, as políticas públicas, o atendimento do enfermeiro e a incorporação de novas rotinas no serviço.

## Resumen

**Objetivo:**

analizar la prevalencia de tuberculosis, coronavirus, condiciones crónicas y vulnerabilidades en inmigrantes y refugiados en Brasil.

**Método:**

se trata de un estudio transversal, del tipo encuesta electrónica, realizado con migrantes internacionales durante la pandemia de COVID-19. Para el análisis se aplicó estadística descriptiva, con cálculo de medidas de posición y dispersión. En cuanto a las variables categóricas, se estimaron las frecuencias relativas y absolutas.

**Resultados:**

participaron del estudio 553 inmigrantes y refugiados, la prevalencia de tuberculosis era del 3,07%, de COVID-19 del 7,2% y de condiciones crónicas del 27,3%. Entre las vulnerabilidades, el 32% reportó desempleo, el 37,6% emigró a Brasil por la situación social de su país y el 33,6% vivía en un asilo o albergue.

**Conclusión:**

la tuberculosis, las enfermedades crónicas y el COVID-19 fueron más prevalentes en inmigrantes y refugiados que en la población general. Por tratarse de una población que aún tiene grandes dificultades para acceder a los servicios de salud y sistemas de protección social, el estudio contribuirá, con base en la evidencia, a las políticas públicas, la atención de enfermería y la incorporación de nuevas rutinas en el servicio.

**Highlights**

(1) High prevalence of tuberculosis and comorbidities among migrants and refugees.

(2) Social vulnerability in migrants and refugees.

(3) Nurses’ training to serve migrants and refugees.

(4) Public policies for migrants’ and refugees’ inclusion in and accessibility to the SUS

## Introduction

Nearly 281 million people are outside their places of origin^1^, including international migrants who chose to live in another country, mainly for economic reasons and refugees who were forced to migrate due to situations related to race, religion, nationality, belonging to a particular social group, political opinion, violation of human rights and armed conflicts^2^.

There are approximately 1.5 million international migrants and 68,000 refugees in Brazil^3^. The migratory process is a complex phenomenon whose impact on health depends on the transportation means that were used, on pathogenic or environmental exposures along the transit routes and, also, on the local and destination epidemiological indicators^4^. This set of factors potentiate the risk of acquiring certain diseases such as tuberculosis (TB)^5^. 

Other important circumstances can influence the illness process of this population segment, such as abandoning the search for health services for diagnosis and/or treatment of diseases hindered by several factors like language, culture and lack of knowledge of the rights in the country in which they settled, among others^4^ ^,^ ^6^, a situation that was aggravated during the coronavirus disease 2019 (COVID-19) pandemic^7^. 

The impact of migration on the health situation of this population segment influences both the emergence of infectious diseases and of chronic non-communicable ones^7^ and nurses play an important role in providing health to populations through the Unified Health System (*Sistema* Único *de Saúde*, SUS) that supports both Brazilians and non-Brazilians.

For the study, it is hypothesized that infectious and chronic diseases are more prevalent in the migrant population than in the general population due to the situation of vulnerability and that they were intensified in the COVID-19 pandemic^1^. 

Considering the difficulty in this population’s access to health services and, therefore, the services’ lack of knowledge of their conditions, including TB, COVID-19, chronic conditions and vulnerabilities, the study is of high importance for public policies and service organizations for equality in the care of these populations.

No other nationwide studies with this population segment and objective were identified, revealing an important knowledge gap. Therefore, it was sought to analyze the prevalence of tuberculosis, coronavirus, chronic conditions and vulnerabilities among migrants and refugees in Brazil.

## Method

### Study design

This is a cross-sectional study of the electronic survey type conducted with migrants and refugees; it was developed in compliance with the guidelines set forth in the Checklist for Reporting Results of Internet E-Surveys guidelines and Strengthening the Reporting of Observational Studies in Epidemiology (STROBE) for cross-sectional studies^8^.

### Study setting 

Brazil has been experiencing an intense wave of migrants and refugees from Venezuela, who have arrived mainly through the northern border of the state of Roraima^9^. Up to 2018, the refugee agency (UNHCR) verified the arrival of 77,885 people in Brazil, of which 8,863 came from Syria, Colombia, Angola and the Democratic Republic of Congo, among other countries. 

### Study population and sample design

The study population consisted of migrants and/or refugees who declared to be foreigners, living in Brazil and aged under 18 years old. The exclusion criteria were being Brazilian and not migrants. In order to reduce the possibility of biases, the migrants’ and refugees’ mother tongue (Spanish) was considered in addition to Portuguese. No restrictions were imposed on the sample. All the respondents during the collection period were included in data analysis. 

As for the sample size, considering that in order to have an expected proportion of 1% as minimum prevalence of chronic conditions (including TB) and 3% as alternative hypothesis, a minimum sample of 286 respondents was estimated for 80% power and 95% significance level. 

### Measuring instrument

The instrument used was a validated questionnaire consisting of closed questions in a language that was accessible to the study population. The instrument validation process was carried out by specialists from the Brazilian Network on Tuberculosis Research (REDE-TB) and from the Epidemiology Laboratory of the Federal University of Espírito Santo (LAB-EPI). 

Semantic validation to assess if the items were compatible with the migrants and refugees was performed in this phase, in addition to legitimizing the content. The aforementioned questionnaire was evaluated regarding its sufficiency using the Delphi Technique, considering classic studies^4^ ^,^ ^10^, having its original version in Portuguese, with translation into Spanish and back-translation into Portuguese, with reliability and agreement analysis by experts in the area, native speakers of their languages.

Due to the characteristics of this population, with difficulty accessing health services and without being included in the official records in Brazil, mainly with regard to those in irregular situations and also due to secrecy, recruitment of the participants was carried out using a non-probabilistic technique, of sequential sampling, with participants being included as they were located and agreed to participate in the study^11^.

### Data collection 

As a disclosing strategy, in order to minimize and allow for greater sample coverage, digital media (Instagram, Facebook, Twitter, etc.), hosting on the LAB-EPI and REDE-TB website and support from non-governmental organizations that work with migrants and refugees in Brazil to disseminate the survey. After being informed about the research and accessing the link to read and agree with the Free and Informed Consent Form (FICF), the participants were granted access to the questionnaire. In case of doubts, there was the possibility of solving them by contacting the lead researcher.

Data collection took place from August 17^th^ to October 30^th^, 2020, defining a two-month deadline to receive the questionnaire in order to reach the target population. This period of time was determined according to the non-governmental organizations that worked with the migrants and refugees. The questionnaires in the Web Survey type were hosted in the Research Electronic Data Capture (REDCap) platform belonging to the Federal University of Espírito Santo (*Universidade Federal do Espírito Santo*, UFES). 

Access to the questionnaire was through tablets, smartphones and computers, according to the migrants’ and refugees’ availability, with Internet access to answer the instrument. The team invested on communication and marketing of the project, considering the reference literature in the area of electronic and/or epidemiological surveys based on the Internet^12^ ^-^ ^13^. The mean time to answer the questionnaire was from 20 to 30 minutes.

### Variables under study 

The questionnaire consisted of sociodemographic information; current and past health status (morbidities such as chronic conditions, including TB); habits and lifestyles; situation of social vulnerability; motivation for migration; social support networks and social protection, including access and/or accessibility to health services. In order to organize the results, the variables were categorized into eight broad topics:

Personal data: gender (female and male), race/skin color/ethnicity (white, black, yellow, brown, indigenous, Creole, I prefer not to answer, other), marital status (single, married/in a stable relationship, separated/divorced, widowed, I prefer not to answer) and state and municipality of residence. In relation to the classification used for the “self-declared race/skin color”, it was decided to encompass all the *Instituto Brasileiro de Geografia e Estatística* (IBGE) categories plus “Creole”. In Venezuela, ‘’Creole’’ refers to any non-indigenous individual^14^. 

Migration data: country of birth, naturalized Brazilian (no, yes, I prefer not to answer), how long ago did you move to Brazil (less than 6 months, from 6 months to less than 1 year, from 1 year to less than 2 years, from 2 years to less than 3 years, 3 years or more, I don’t remember), reason for moving to Brazil [work, study, family, situation of vulnerability in the country of origin, health, I prefer not to answer, other (open answer)], how long do you intend to live in Brazil [permanently, end of employment contract, end of studies, improvement in the situation in the country of origin, improvement in health, I prefer not to answer, other (open answer)]. 

Vulnerable populations: do you fall into any situation of vulnerability [I do not fall into any situation of vulnerability, deprived of freedom, living on the street, indigenous, health professionals, resident of an asylum/shelter/hostel, I prefer not to answer, other (open answer)].

Socioeconomic data: instruction/schooling level (did not study, up to 4 years, between 5 and 8 years, between 9 and 11 years, incomplete higher education, complete higher education, graduate, I prefer not to answer), occupation [student, unemployed, formal worker, informal worker, worker and student, I prefer not to answer, other (open answer)], was your family income reduced during the COVID-19 pandemic (no, yes, I prefer not to answer, I don’t know), where do you live [own house, rented house, relatives’/friends’ house, asylum/shelter/hostel, student accommodation, no fixed place of residence, deprived of freedom, living on the street, other (open answer)], including you, how many people sleep in the same room (up to 2, from 3 to 5, from 6 to 8, from 9 to 12, from 13 to 15, more than 15, I prefer not to answer), do you and/or your family send money to your country of origin (no, yes, I prefer not answer). Regarding the “formal worker” option, it corresponded to those who had a formal contract and the inclusion of “students” in the occupation category was to expand the analyses, considering the change in the profile of migrations and the mean age of migrants and refugees in Brazil^1^. 

Tuberculosis: were you diagnosed with TB (no, yes, I prefer not to answer, I don’t know), how long ago (less than 3 years, 3 years or more, I don’t know, I prefer not to answer), where was the TB diagnosis made (country of origin, Brazil), did you receive TB treatment (no, yes, I prefer not to answer), where was the TB treatment performed (country of origin, Brazil), did you complete the TB treatment (no, yes, I don’t remember, I prefer not to answer), if not, why [lack of money for treatment costs, medication side effects, moving, distance from the health service, could not access the health services, other (open answer)], did you have contact with someone diagnosed (no, yes, I prefer not to answer), how long ago did this contact take place (less than 3 years, 3 years or more, I don’t know, I prefer not to answer), where did this contact take place (country of origin, Brazil), were you evaluated by a health professional after this contact (no, yes, I prefer not to answer).

Health history: were you diagnosed with any chronic disease (no, yes, I prefer not to answer), which chronic diseases: alcoholism (no, yes, I prefer not to answer), how long ago was it diagnosed (less than 3 years, 3 years or more, I don’t know, I prefer not to answer), diabetes (no, yes, I prefer not to answer), how long ago was it diagnosed (less than 3 years, 3 years or more, I don’t know, I prefer not to answer), human immunodeficiency virus (HIV) (no, yes, I prefer not to answer), how long ago was it diagnosed (less than 3 years, 3 years or more, I don’t know, I prefer not to answer), hypertension (no, yes, I prefer not to answer), how long ago was it diagnosed (less than 3 years, 3 years or more, I don’t know, I prefer not to answer), depression (no, yes, I prefer not to answer), how long ago was it diagnosed (less than 3 years, 3 years or more, I don’t know, I prefer not to answer), other diseases for which you undergo treatment that were not mentioned [no, yes, I don’t know, I prefer not to answer, others (open answer)].

COVID-19: did you have COVID-19 (no, yes, I don’t know, I prefer not to answer), were you evaluated by a health professional (no, yes, I don’t know, I prefer not to answer), did you need to be hospitalized due to COVID-19 (no, yes, I don’t know, I prefer not to answer), did you seek some other health care facility because of COVID-19 (no, yes, I don’t know, I prefer not to answer), were you tested for COVID-19 (no, yes, I don’t know, I prefer not to answer), what was the result (negative, positive, I prefer not to answer), did someone who lives with you have COVID-19 (no, yes, I don’t know, I prefer not to answer), was that person tested for COVID-19 (no, yes, I don’t know, I prefer not to answer), result (negative, positive, I don’t know, I prefer not to answer), did you maintain social distancing during the COVID-19 pandemic period (all the time, much of the time, part of the time, a little, no), you consider that your work/study during the COVID-19 pandemic period was (not affected, slightly affected, affected, greatly affected, completely affected, not applicable), did you think about returning to your country of origin because of the COVID-19 pandemic (all the time, most of the time, part of the time, a little, no), when possible, you will return to your home country because of the COVID-19 pandemic (no, yes, I don’t know, I prefer not to answer), did a family member or friend in your home country have COVID-19 (no, yes, I don’t know, I prefer not to answer), did you receive any governmental benefit during the COVID-19 pandemic (no, yes, I prefer not to answer).

Insurance and health system: do you have any type of private health/medical insurance plan (no, yes, I don’t know, I prefer not to answer), have you used or do you use the SUS (no, yes, I don’t know, I prefer not to answer).

### Data analysis

Using the resources from the REDCap platform allowed monitoring the answers and creating a database in a safe and confidential environment. Better data quality was ensured through this technology, with a reduction of variables with unanswered information and validation of these data by field supervisors, thus reducing measurement bias. Descriptive statistics was applied for data analysis, with calculation of position (mean and median) and dispersion (standard deviation) measures. The relative and absolute frequencies of the categorical variables were estimated by means of the STATA 14.0 statistical program. The statistical analyses were performed in the *R 4.1.1* statistical software.

### Ethical aspects

The study observed all the ethical aspects in full compliance with Resolutions No. 466 of 2012 and No. 510 of 2016 of the National Council of Research Ethics (*Conselho Nacional de* Ética *em Pesquisa*, CONEP). The study was approved by the Research Ethics Committee (*Comitê de* Ética *em Pesquisa*, CEP) of the Federal University of Espírito Santo (UFES) under opinion nº 3,953,347, by the CONEP, and by the Pan American Health Organization Ethics Review Committee (PAHOERC) under nº 0204.03. All participants agreed to the FICF available in electronic format also with consent in an electronic record, with a version of this document sent to the participant’s email address. The page was automatically closed for those that did not agree.

## Results

The study participants were 533 migrants and/or refugees: 426 respondents for the Portuguese version of the survey and 127 for its Spanish counterpart. Table 1 shows the main characteristics of the study population. Regarding gender, the majority are female (58.2%), self-declared as mixed race/skin color (41.0%) and with higher education or graduate studies (40.9%), single marital status (58.2%), not naturalized Brazilian (92.6%) and for more than three years in the country (31.6%). 

**Table 1:** Sociodemographic characteristics of the migrants and refugees (n = 553). Brazil, 2020

Variables	n	%
*Gender*		
Female	322	58.2%
Male	231	41.8%
*Self-declared race/skin color*		
Brown	227	41.0%
White	168	30.4%
Black	85	15.4%
Indigenous	20	3.6%
Creole	16	2.9%
Other	16	2.9%
Unknown	11	2.0%
Asian	10	1.8%
*Schooling*		
Complete Higher Education	142	25.7%
Incomplete Higher Education	101	18.3%
Between nine and eleven years of study	91	16.5%
Graduate studies	84	15.2%
Between five and eight years of study	70	12.7%
Up to four years of study	30	5.4%
No studies	21	3.8%
I prefer not to answer	13	2.4%
Unknown	1	0.2%
*Marital status*		
Single	286	51.8%
Married/Stable union	206	37.3%
Separated/Divorced	34	6.1%
Widowed	18	3.3%
Unknown	9	1.6%
*Employment status*		
Unemployed	177	32.0%
Informal worker	155	28.0%
Student	76	13.7%
Formal worker	83	15.0%
Other	25	4.5%
I prefer not to answer	19	3.4%
I work and study at the same time	15	2.7%
Unknown	3	0.5%
*Naturalized*		
No	512	92.6%
Yes	35	6.3%
I prefer not to answer	6	1.1%
*Vulnerable population*		
Unknown	7	1.3%
Living on the street	3	0.5%
Deprived of freedom	2	0.4%
Indigenous	1	0.2%
*Non-vulnerable population*	540	97.6%
*Housing situation*		
Rented house	256	46.2%
Asylum/Shelter/Hostel	186	33.6%
Relatives’ or friends’ house	51	9.2%
Own home	25	4.5%
I prefer not to answer	15	2.0%
No fixed place of residence	7	1.3%
Unknown	13	2.4%
Student accommodation	-	-
I’m living on the street	-	-
*Time living in Brazil*		
3+ years	175	31.6%
More than 1 year but less than 2 years	152	27.5%
More than 6 months but less than 1 year	100	18.0%
More than 2 years but less than 3 years	87	15.7%
Less than 6 months	22	4.0%
I don’t remember	17	3.1%

As for the motivation to migrate to Brazil, most of the migrants and refugees justified it due to the situation of vulnerability in their country of origin (37.6%), followed by looking for work (22.8%) and studying (14.5%). Of the states of residence, the majority (51.4%) live in Roraima, followed by São Paulo (9.6%). It was also observed that the majority intends to stay in the country (49.5%). Table 2 shows that practically 90.2% of them have no health plan and/or private health insurance and that 84.6% report using the Unified Health System (SUS). 

**Table 2:** Reason for moving, state of residence of the migrants and refugees, length of stay in Brazil and health system coverage (n=553). Brazil, 2020

Variables	n	%
*Reason for moving to Brazil*		
Situation of vulnerability in their country of origin	208	37.6%
Work	126	22.8%
Study	80	14.5%
Family	72	13.0%
Health	26	4.7%
I prefer not to answer	24	4.3%
Other	15	2.7%
Unknown	2	0.4%
*Migrant’s state of residence*		
Roraima/RR	284	51.4%
São Paulo/SP	53	9.6%
Espírito Santo/ES	50	9.0%
Others	32	5.8%
Distrito Federal/DF	28	5.1%
Rio Grande do Sul/RS	24	4.3%
Rio de Janeiro/RJ	17	3.1%
Amazonas/AM	13	2.4%
Minas Gerais/MG	13	2.4%
Paraná/PR	12	2.2%
Bahia/BA	11	2.0%
Rio Grande do Norte/RN	8	1.4%
Santa Catarina/SC	8	1.4%
*Estimated length of stay in Brazil*		
Permanently	274	49.5%
Improvement in the situation in the country of origin	140	25.3%
I prefer not to answer	58	10.5%
End of studies	43	7.8%
Other	18	3.3%
Improvement in health status	10	1.8%
End of employment contract	7	1.3%
Unknown	3	0.5%
*Health plan/Private health insurance*		
No	499	90.2%
Yes	51	9.2%
I prefer not to answer	3	0.5%
*Uses the Unified Health System (SUS)*		
Yes	468	84.6%
No	76	13.7%
Unknown	9	1.6%

Through the Web Survey, the migrants and refugees were asked about their TB history and regarding contacts with index cases, as shown in Table 3. 3.07% prevalence of self-reported TB cases was observed and it is also reported that 11.2% of the participants had contact with someone infected by TB, and only 6% were evaluated by a health professional. 

Of the 17 respondents that stated having being diagnosed with TB, 53.3% have had the disease for over 3 years. Slightly more than 66% were diagnosed in Brazil and all of them stated having finished the treatment and being cured.

**Table 3:** Situation of self-reported tuberculosis among migrants and refugees (n = 553). Brazil, 2020

	Yes	%	No	%
Contact with someone infected by TB*	62	11.2%	491	88.8%
Evaluated by a health professional after contact with a TB* index case	33	6.0%	520	94.0%
TB* diagnosis	17	3.07%	536	96.9%

*TB = Tuberculosis

As for the self-reported health conditions, 27.3% prevalence of some chronic condition was observed in Table 4, with hypertension (28.5%), diabetes (21.2%), depression (14.6%) and all other chronic conditions (25.2%) standing out in decreasing order of frequency. 5.3% self-reported prevalence of HIV and of alcoholism was also identified. 

**Table 4:** Self-reported health conditions among the migrants and refugees (n = 553). Brazil, 2020

Health conditions	n	%
No self-declared chronic condition	402	72.7%
Some chronic condition	151	27.3%
Hypertension	43	28.5%
Other condition	38	25.2%
Diabetes	32	21.2%
Depression	22	14.6%
Alcoholism	8	5.3%
HIV^*^	8	5.3%

* HIV = Human Immunodeficiency Virus

Considering the pandemic situation, Table 5 shows prevalence of the disease among the migrants and refugees: 7.2% of the respondents were infected by the SARS-CoV-2 virus and 5.2% were examined by a health professional regarding the self-reported disease. Only 22.6% were tested for COVID-19 and 5.8% stated having tested positive for the disease. There was 9.9% prevalence of contacts with people affected by COVID-19; among these contacts, 6.5% reported having undergone the test for the disease, with 6% of positive results. On the other hand, 53.5% received governmental support during the COVID-19 pandemic.

**Table 5:** Impact imposed by COVID-19 among migrants and refugees (n = 553). Brazil, 2020

COVID-19 domain	n	%	n	%
Had COVID-19^*^	40	7.2%	513	92.8%
Evaluated by a health professional regarding the self-reported disease	29	5.2%	524	94.8%
Need to be hospitalized due to COVID-19^*^	2	0.4%	551	99.6%
Tests performed to confirm COVID-19^*^	125	22.6%	428	77.4%
Positive result in the COVID-19^*^ test	32	5.8%	521	94.2%
Person that lives with a migrant and had COVID-19^*^	55	9.9%	498	90.1%
The contact was tested for COVID-19^*^	36	6.5%	517	93.5%
The contact was positive for COVID-19^*^	33	6.0%	520	94.0%
Received governmental support during the COVID-19^*^ pandemic	296	53.5%	257	46.5%

* COVID-19 = Coronavirus Disease 2019

## Discussion

The objective of the study was to analyze the prevalence of TB, COVID-19, chronic conditions and vulnerabilities among migrants and refugees. The following was observed in this population segment: the majority belonged to the female gender (58.2%), they were of brown/black/creole race/skin color (58.9%) and they had Complete Higher Education (25.7%). Prevalence of TB (3.07%), hypertension (28.5%), diabetes (21.2%) and COVID-19 (7.2%), which corroborates the study hypothesis that, considering the data presented, prevalence would be higher than in the general population.

The sociodemographic characteristics presented differ in some points from other studies conducted with migrants and refugees, with emphasis on the fact that the majority belongs to the female gender, has Complete Higher Education and does not self-report being in social vulnerability conditions, as the online survey was able to capture a population group with a different reality regarding massive migration to Brazil, but which is similar in terms of brown and/or black and/or Creole race/skin color, predominant among the participants^15^ ^-^ ^16^. 

In Brazil, a classic study estimated that 1% of the population would present respiratory symptoms for TB, 7.4% prevalence of diabetics and 24.5% of hypertensives, numbers lower than those revealed in the study^17^. 

Another study, conducted in the United States, on TB and other health conditions in newly arrived migrants, identified an even higher prevalence of TB (25%), with 34% presenting some comorbidity, with hypertension, obesity, hepatitis and diabetes mellitus among the most common^5^. It is worth mentioning that, probably, the numbers found have a limitation because they are self-reported, taking into account the possible barriers in access to the health system that can prevent migrants and refugees from knowing their current health condition in Brazil^18^, mainly in the COVID-19 pandemic context in which the survey was carried out.

There are still no studies on the prevalence of COVID-19 among migrants and refugees. High prevalence of COVID-19 was verified in this study. The data were collected before the second and third COVID-19 waves in Brazil. It is known that the country did not adopt the mass testing global recommendation^19^ and that the estimated prevalence is biased without such testing. In addition to that, this study was conducted before the gamma and omicron waves in Brazil, which were responsible for the highest incidence values regarding cases of the disease^20^. 

Most of the interviewees stated having complied with social isolation all or most of the time and reported having their family income affected during the period. In order to mitigate this harm, there was predominance of people who stated having received governmental support and having resorted to the SUS. According to the findings, 46.5% of the migrants and refugees reported that they received governmental aid during availability of the resource, which, in a way, is below the 60% identified in the study, among those unemployed and/or with informal work, target population to receive the benefit. 

It was also observed that 33.6% were living in hostels and/or shelters, that 62.4% did not consider themselves in conditions of vulnerability and that 90.2% did not have health insurance. Although the perception of social vulnerability is subjective, the United Nations (UN) and the UNHCR consider that migrants and refugees are socially vulnerable^5^ ^,^ ^20^. In the present study, the significant number of people receiving emergency aid draws the attention, totaling almost half of the sample under study. 

It is believed that, during displacements and in search of resettlement in another country, migrants and refugees go through stressful situations, with difficulties communicating in another language, as well as regarding housing and working conditions. These situations expose healthy individuals to pathogens, climate and environmental changes along transit routes, epidemiological indicators of the place of origin and of the destination countries^5^ ^,^ ^20^.

In the Collective Health Nursing scope, it is necessary to observe the geopolitical territory of production, social reproduction and work in health for a transformation of the epidemiological profiles of these populations which are targets for global discussions, due to the impacts caused by migrations and demand knowledge and competences from nurses to recognize the health needs and cope with the diseases to which they are exposed^21^.

In relation to their work status, 32% stated being unemployed in Brazil during the pandemic. It must be said that the Emergency Aid Program was the main income-related measure adopted by the Brazilian federal government during the pandemic, with exceptional social protection measures to be adopted, granting R$ 600.00 (US$ 114,28), initially for three months, to informal workers, unemployed and self-employed people and individual micro-entrepreneurs and R$ 1,200.00 (US$ 228,57) for single-parent female families^22^.

It was noticed that, due to the bureaucracy required by this income transfer system and as a result of the major difficulty of this population in holding the necessary documents to request the aid, this group was at a disadvantage in terms of accessibility to the Program, which places them in a situation of greater vulnerability and inequality. 

Among the study limitations, the prevalence of TB, COVID-19 and other morbidities was self-reported by the study participants and may not represent the migrants’ health situation in its entirety. The electronic survey, made available on the Internet, may not have been accessed by the most socially vulnerable migrants and refugees, as they do not have access to the Internet or to electronic devices. In addition, language may have been a barrier for those that do not master Portuguese or Spanish. 

It is believed that the data presented in this study/survey may provide subsidies to optimize service provision by health professionals, mainly nurses, in order to enable migrants and refugees to access the health system more effectively and resolutely; in addition to the development of effective actions in the field of education in health, as well as assisting in the elaboration of specific health policies for this population, ensuring a safety net. 

## Conclusion

TB, diabetes mellitus, hypertension and COVID-19 presented higher prevalence values among migrants and refugees than in the general population. Although the health conditions are self-reported, it was possible to evidence the health situation, with emphasis on some infectious diseases and/or chronic conditions, on the vulnerability of these groups in terms of housing, and on the use of income transfer programs. 

As this is a population segment that still has significant difficulty accessing health services and social protection systems, the study will serve as an evidence base for public policies that induce inclusion, accessibility and the rights of migrants and refugees.
